# Blue Light Improves Photosynthetic Performance during Healing and Acclimatization of Grafted Watermelon Seedlings

**DOI:** 10.3390/ijms22158043

**Published:** 2021-07-28

**Authors:** Moein Moosavi-Nezhad, Reza Salehi, Sasan Aliniaeifard, Georgios Tsaniklidis, Ernst J. Woltering, Dimitrios Fanourakis, Krystyna Żuk-Gołaszewska, Hazem M. Kalaji

**Affiliations:** 1 Department of Horticultural Sciences, Campus of Agriculture and Natural Resources, University of Tehran, Karaj P.O. Box 31587-77871, Iran; Moein.moosavi@ut.ac.ir; 2 Photosynthesis Laboratory, Department of Horticulture, Aburaihan Campus, University of Tehran, Tehran P.O. Box 33916-53755, Iran; 3 Laboratory of Vegetable Crops, Institute of Olive Tree, Subtropical Plants and Viticulture, Hellenic Agricultural Organization ‘ELGO DIMITRA’, 73100 Chania, Greece; tsaniklidis@elgo.iosv.gr; 4 Wageningen Food & Biobased Research, Bornse Weilanden 9, 6708 WG Wageningen, The Netherlands; Ernst.Woltering@wur.nl; 5 Horticulture & Product Physiology, Wageningen University, Droevendaalsesteeg 1, 6708 PB Wageningen, The Netherlands; 6 Laboratory of Quality and Safety of Agricultural Products, Landscape and Environment, Department of Agriculture, School of Agricultural Sciences, Hellenic Mediterranean University, Estavromenos, 71004 Heraklion, Greece; dimitrios.fanourakis82@gmail.com; 7 Department of Agrotechnology and Agribusiness, Faculty of Agriculture and Forestry, University of Warmia and Mazury in Olsztyn, ul. Oczapowskiego 8, 10-718 Olsztyn, Poland; kzg@uwm.edu.pl; 8 Department of Plant Physiology, Institute of Biology, Warsaw, University of Life Sciences SGGW, 02-776 Warsaw, Poland; hazem@kalaji.pl; 9 Institute of Technology and Life Sciences—National Research Institute, Falenty, Al. Hrabska 3, 05-090 Raszyn, Poland

**Keywords:** chlorophyll fluorescence imaging, healing and acclimatization, light quality, O–J–I–P-transient, vegetable grafting

## Abstract

To investigate the importance of light on healing and acclimatization, in the present study, grafted watermelon seedlings were exposed to darkness (D) or light, provided by blue (B), red (R), a mixture of R (68%) and B (RB), or white (W; 35% B, 49% intermediate spectra, 16% R) LEDs for 12 days. Survival ratio, root and shoot growth, soluble carbohydrate content, photosynthetic pigments content, and photosynthetic performance were evaluated. Seedling survival was not only strongly limited in D but the survived seedlings had an inferior shoot and root development, reduced chlorophyll content, and attenuated photosynthetic efficiency. RB-exposed seedlings had a less-developed root system. R-exposed seedlings showed leaf epinasty, and had the smallest leaf area, reduced chlorophyll content, and suppressed photosynthetic apparatus performance. The R-exposed seedlings contained the highest amount of soluble carbohydrate and together with D-exposed seedlings the lowest amount of chlorophyll in their scions. B-exposed seedlings showed the highest chlorophyll content and improved overall PSII photosynthetic functioning. W-exposed seedling had the largest leaf area, and closely resembled the photosynthetic properties of RB-exposed seedlings. We assume that, during healing of grafted seedlings monochromatic R light should be avoided. Instead, W and monochromatic B light may be willingly adopted due to their promoting effect on shoot, pigments content, and photosynthetic efficiency.

## 1. Introduction

In vegetable crops, grafting has been widely employed to improve fruit quality, as well as to achieve better yields under biotic (e.g., soil-borne diseases) and abiotic (e.g., drought, salinity, non-optimum temperatures) stresses [1]. It is a widely applied agricultural practice on species of the *Cucurbitaceae* and *Solanaceae* families [2,3], and in particular in tomato and watermelon, with the latter showing the lowest survival rates [4].

For gaining the advantages of grafting, high-quality seedlings ought to be produced [5]. This necessitates a successful connection between rootstock and scion, a process that takes place during the healing and acclimatization period [6]. During the first days of this period (healing), the newly-grafted seedlings have no vascular connections, and the scion part often encounters desiccation. Optimizing environmental factors during healing and acclimatization stages is thus vital for seedling production [3]. Healing and acclimatization are generally carried out under shaded plastic-covered tunnels, often referred to as conventional tunnel systems. In these systems, relative air humidity is maintained at high levels to prevent excessive water loss and the resultant scion wilting. Letting aside that direct exposure to natural light is avoided (by shading), light conditions are generally poorly controlled since they are prone to diurnal and seasonal changes [7]. Therefore, among environmental factors, the role of light quality on mediating seedling growth and vigor is currently the most neglected one [8,9]. This topic has been currently addressed by a handful of studies, where different light quality regimes were reported for driving optimal growth during healing and acclimatization [5,6,10]. For instance, monochromatic red (R) light led to optimum grafted tomato seedling quality [5], whereas a spectrum including both blue (B) and R spectral regions was required in pepper [6]. Given the noted interspecific variation in response to light spectrum, seedling quality ought to be individually evaluated in the species of interest before commercial implementation.

Generally, high-quality seedlings are regarded as the ones showing uniformity in greenness, a criterion that is often employed throughout the production-distribution chain (e.g., vegetable nurseries, growers). Instead, yellowing symptoms of scion cotyledon are a clear sign of improper healing and acclimatization. Therefore, assessing the greening in different parts of the grafted seedlings is an index of their vigor [2].

Carbohydrates are the main source of energy in plants, and their abundance is a prerequisite for cell division activity and region differentiation. In this respect, the carbohydrate status is expected to play a major role in the healing of grafted seedlings. This status is related to both photosynthetic activity, and to the available amount of carbohydrates in the scion. Although carbohydrate status has been earlier associated to light quality, the respective effects on seedling growth and vigor have received limited attention [11,12].

*De novo* carbohydrate synthesis through photosynthesis is vital for the grafting success [13]. During healing and acclimatization, environmental conditions may exert a considerable impact on the reactions involved in the photosynthetic process [14]. For instance, the photosynthesis of grafted seedlings may be interrupted by mechanical stress, water deficit in scion, or these two combined. On top of this, seedlings are very sensitive to light stress at this stage. Chlorophyll fluorescence is often utilized for non-invasive evaluation of electron transport system efficiency. Moreover, polyphasic chlorophyll fluorescence induction curves can be employed to investigate the fate of absorbed light energy and knowledge about the structure and function of the photosynthetic apparatus [15,16].

The present investigation was done to gain insight in the effect of light quality during healing and acclimatization of grafted watermelon seedlings on survival, morphology, carbohydrate status, and photosynthetic pigment content in different leaf types. In addition, chlorophyll fluorescence parameters were quantified as well as total biomass to assess the impact of light spectrum on photosynthetic performance. All these parameters are directly associated to the seedling quality. Thus, selecting the most appropriate light quality for healing and acclimatization would pave the way for the expansion of vertical-pattern systems, which rely exclusively on artificial light sources [17,18].

## 2. Results

### 2.1. Survival, Growth and Morphology of Grafted Seedlings

Following grafting and 2 days of light deprivation, seedlings were exposed to either D or different light quality regimes for 12 days prior to sampling. Survival ratio was 26% following exposure to D, whereas all seedlings survived under light irrespective of spectral quality (Table 1). Shoot dry weight, root dry weight, and leaf area per plant were highly suppressed under D, as compared to seedlings exposed to light. Shoot dry weight was not significantly different among seedlings exposed to different light spectra, while root dry weight was significantly lower in RB-exposed seedlings. The treatments with enhanced proportion of blue (i.e., B and W) resulted in taller scions. Among light treatments, leaf area per plant was the lowest in R-exposed seedlings, mainly owing to smaller individual leaf area rather than a lower number of leaves (Table 1).

W-exposed seedlings had the highest SLA, while the lowest one was noted under D. B-, RB- and R-exposed seedlings showed intermediate SLA values. D- and RB-exposed seedlings had the highest LMR, while the lowest one was noted in B- and R-exposed seedlings. Shoot-to-root ratio of D- and RB-exposed seedlings was significantly lower as compared to seedlings exposed to the other light regimes. By using the DQI, as a quality measure, the seedlings exposed to D were of inferior quality, as compared to the ones exposed to light irrespective of quality (Table 1).

**Figure 1:**
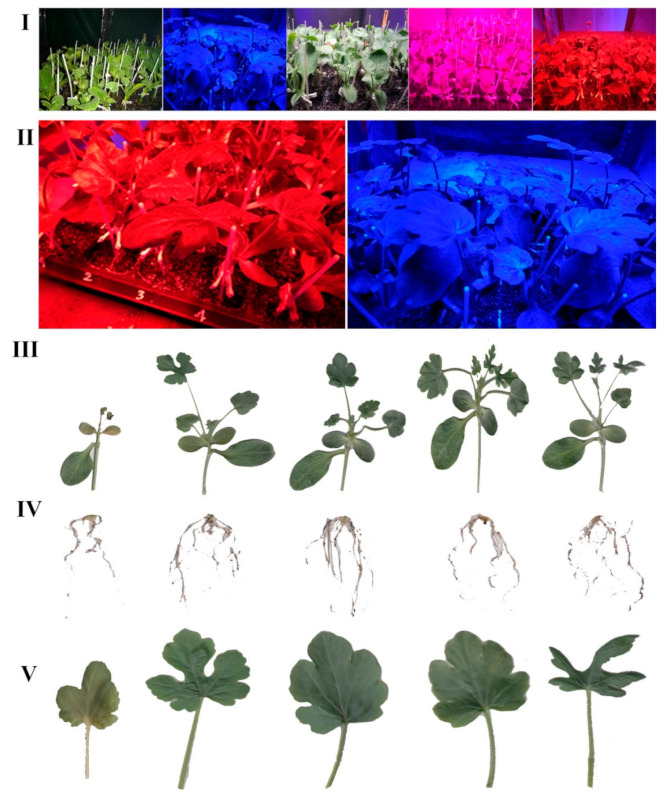
Images of grafted watermelon seedlings following 12 days of exposure to the respective light environment (from left to right: darkness, blue, white, red and blue, red; see spectrum in Supplementary Figure S3). Images include cultivation (**I**); where blue as well as red are magnified in (**II**), shoot (**III**), root (**IV**), and leaf (**V**) levels.

Leaf morphology was also affected by light spectra (Figure 1). Downward curling of leaf margins was only observed in R-exposed seedlings, whereas flattened leaves were apparent in B-exposed seedlings (Figure 1II).

### 2.2. Leaf Soluble Carbohydrates

**Figure 2:**
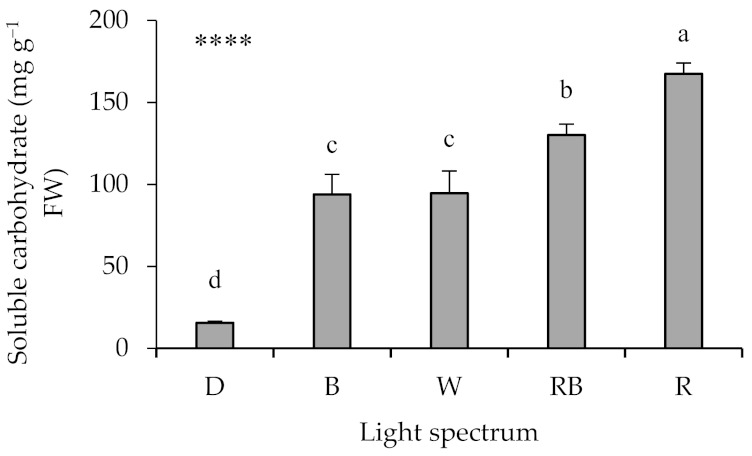
Total leaf carbohydrate content in grafted watermelon seedlings exposed for 12 days to either darkness (D) or different light quality regimes [blue (B), white (W), red and blue (RB), as well as red (R); see spectrum in Supplementary Figure S3]. Photosynthetic photon flux density was 20 ± 1 µmol m^−2^ s^−1^ during the first 2 days, and 50 ± 1 µmol m^−2^ s^−1^ during the following days. Nine plants per treatment were assessed. Different letters indicate that values are significantly different at *p* < 0.01 according to Duncan’s multiple range tests. Bars represent SEM. Significance at the 0.0001 probability level is indicated by ****.

The lowest concentration of soluble carbohydrates was noted in seedlings under D (Figure 2). Among light treatments, the treatments with enhanced proportion of blue (i.e., B and W) had the lowest soluble carbohydrate concentration, while the highest concentration was noted in R-exposed seedling (Figure 2).

### 2.3. Leaf Photosynthetic Pigments

**Figure 3:**
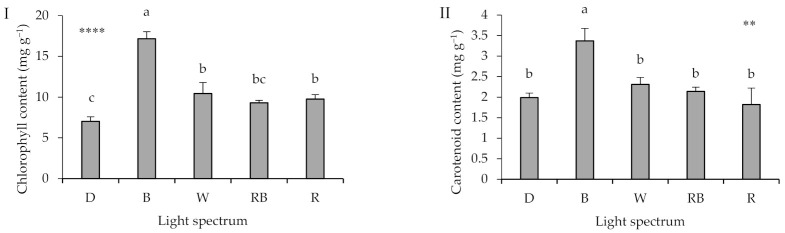

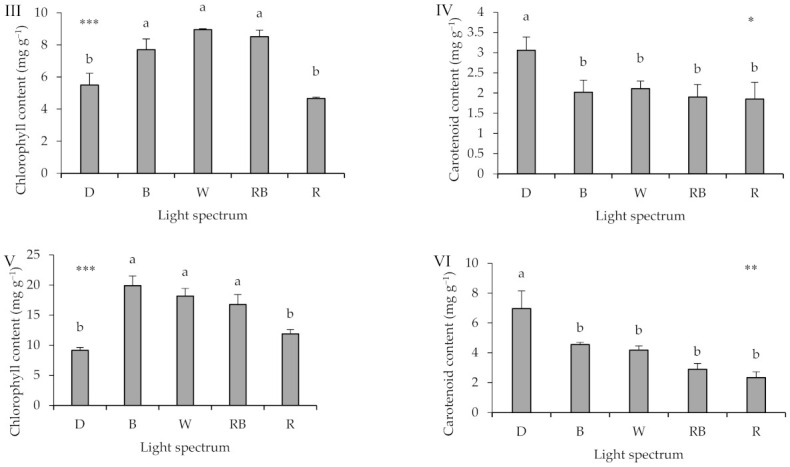
Chlorophyll and carotenoid contents of rootstock cotyledon (**I**, **II**, respectively), scion cotyledon (**III**, **IV**, respectively), and scion leaf (**V**, **VI**, respectively) in grafted watermelon seedlings exposed for 12 days to either darkness (D) or different light quality regimes [blue (B), white (W), red and blue (RB), as well as red (R); see spectrum in Supplementary Figure S3]. Photosynthetic photon flux density was 20 ± 1 µmol m^−2^ s^−1^ during the first 2 days, and 50 ± 1 µmol m^−2^ s^−1^ during the following days. Nine plants per treatment were assessed. Different letters indicate that values are significantly different at *p* < 0.01 according to Duncan’s multiple range tests. Bars represent SEM. Significance at the 0.05, 0.01, 0.001 and 0.0001 probability levels are indicated by *, **, *** and ****, respectively.

Optimum greenness in different parts of grafted seedlings is indicative of proper healing, and is therefore, vital for their marketability. Under this background, pigments concentration was evaluated in rootstock cotyledon, scion cotyledon and scion leaf (Figure 3). The effect of light regime during healing and acclimatization on pigment concentration varied among the tissue under study. In rootstock cotyledon, B-exposed seedlings had increased chlorophyll and carotenoid contents (Figure 3I,II). In both scion cotyledon and leaf, R- and D-exposed seedlings had the lowest chlorophyll content (Figure 3III,V). In scion cotyledons and leaves, carotenoid content was not affected by light environment (Figure 3IV). In both scion cotyledons and leaves, D stimulated carotenoid content, as compared to the light treatments (Figure 3VI).

### 2.4. Chlorophyll Fluorescence Imaging

**Figure 4:**
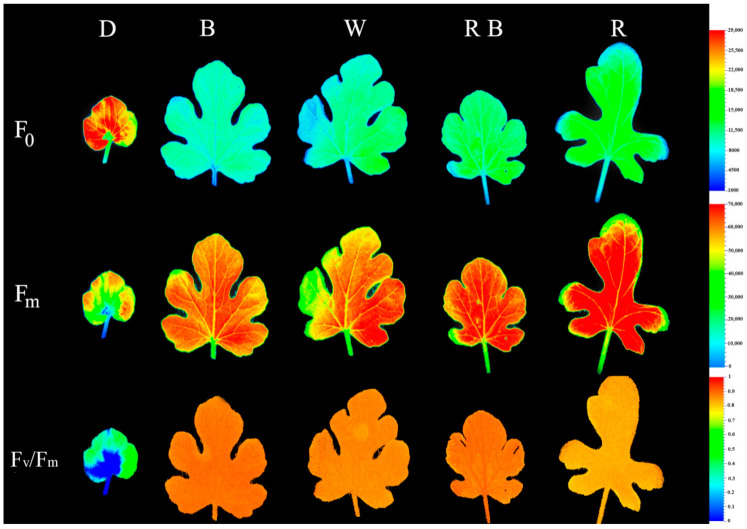
Pseudo-color images of F_0_, F_m_, and F_v_/F_m_ (equations in Table 2) exhibited by leaves sampled from grafted watermelon seedlings exposed for 12 days to either darkness (D) or different light quality regimes [blue (B), white (W), red and blue (RB), as well as red (R); see spectrum in Supplementary Figure S3]. Photosynthetic photon flux density was 20 ± 1 µmol m^−2^ s^−1^ during the first 2 days, and 50 ± 1 µmol m^−2^ s^−1^ during the following days. Results from all nine plants were similar, representative images are shown.

The effect of light environment during healing and acclimatization on overall photosynthetic functionality was assessed by evaluating the spatial pattern of fluorescence emission through pseudo-color images of F_0_, F_m_, and F_v_/F_m_ (Figure 4; equations in Table 2). Exposure to D led to the highest F_0_ and the lowest F_v_/F_m_, as compared to the other treatments. Among light treatments, R-exposed seedlings had the highest F_0_, F_m_ and the lowest F_v_/F_m_. B-exposed seedlings achieved the highest F_v_/F_m_ value.

### 2.5. Polyphasic Chlorophyll Fluorescence Transient (OJIP) Evaluation

Transient chlorophyll fluorescence analysis was recorded in leaves expanded under different light regimes. Minor changes were detected in the OJIP steps of B-, W-, and RB-exposed seedlings, whereas striking changes were observed under either D or R regimes (Supplementary Figure S1). Under D, fluorescence intensity of I and P steps was the lowest as compared to the other treatments, while fluorescence intensity of the O step was the highest. Under R light, all steps of the OJIP graph (F_0_, F_J_, F_I_, and F_m_) were increased as compared to the other light quality regimes.

**Figure 5:**
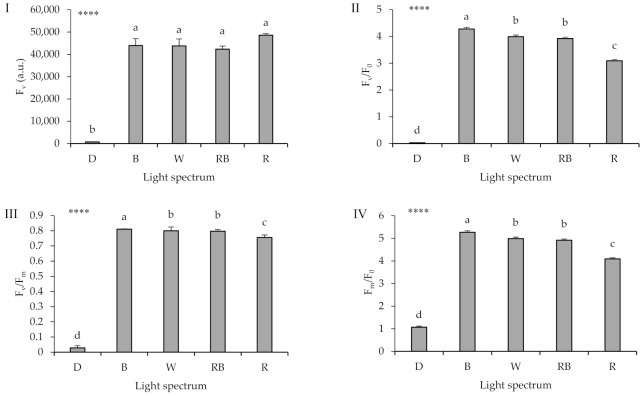
Chlorophyll a fluorescence of the OJIP-test including F_v_ (**I**), F_v_/F_0_ (**II**), F_v_/F_m_ (**III**) and F_m_/F_0_ (**IV**); equations and explanations in Table 2) from leaves sampled from grafted watermelon seedlings exposed for 12 days to either darkness (D) or different light quality regimes [blue (B), white (W), red and blue (RB), as well as red (R); see spectrum in Supplementary Figure S3]. Photosynthetic photon flux density was 20 ± 1 µmol m^−2^ s^−1^ during the first 2 days, and 50 ± 1 µmol m^−2^ s^−1^ during the following days. Nine plants per treatment were assessed. Different letters indicate that values are significantly different at *p* < 0.01 according to Duncan’s multiple range tests. Bars represent SEM. Significance at the 0.0001 probability level is indicated by ****.

Seedling exposure to D led to a dramatic decrease in the values of F_v_, F_v_/F_0_, F_v_/F_m_, and F_m_/F_0_ (Figure 5; equations and explanations in Table 2). Among light treatments, B led to the highest F_v_/F_0_, F_v_/F_m_, and F_m_/F_0_ values, while R light led to the lowest values in these parameters.

**Figure 6:**
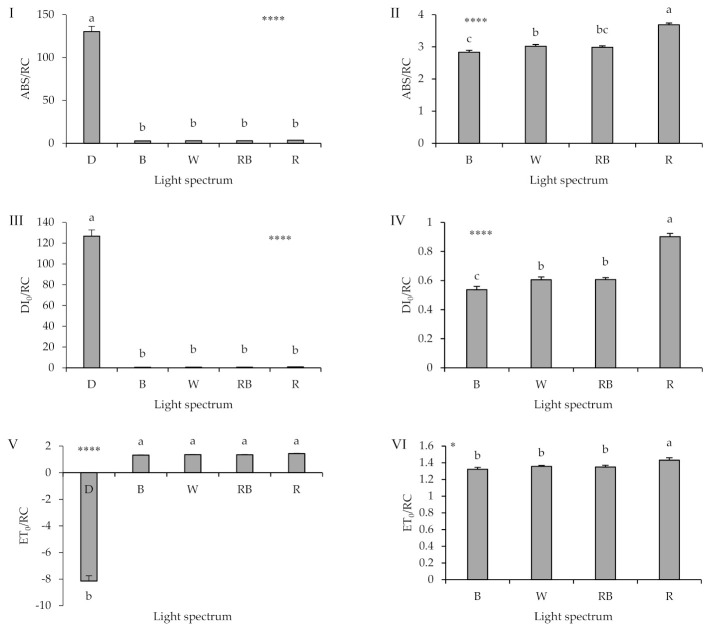
Specific energy fluxes per reaction center (RC) for energy absorption (ABS/RC; **I**, **II**), dissipated energy flux (DI_0_/RC; **III**, **IV**), electron transport flux (ET_0_/RC; **V**, **VI**); equations and explanations in Table 2) from the fluorescence transient exhibited by leaves sampled from grafted watermelon seedlings exposed for 12 days to either darkness (D) or different light quality regimes [blue (B), white (W), red and blue (RB), as well as red (R); see spectrum in Supplementary Figure S3]. Photosynthetic photon flux density was 20 ± 1 µmol m^−2^ s^−1^ during the first 2 days, and 50 ± 1 µmol m^−2^ s^−1^ during the following days. The Panels II, IV, and VI (on the right) represent the same data as the Panels I, III, and V (on the left), besides the darkness treatment which was excluded. Nine plants per treatment were assessed. Different letters indicate that values are significantly different at *p* < 0.01 according to Duncan’s multiple range tests. Bars represent SEM. Significance at the 0.05, and 0.0001 probability levels is indicated by * and ****, respectively.

A striking difference in the parameters related to specific energy fluxes per reaction center (RC) (DI_0_/RC, ABS/RC, and ET_0_/RC; equations and explanations in Table 2) was noted between seedlings exposed to either D or light irrespective of quality (Figure 6). DI_0_/RC and ABS/RC increased by up to two orders under D. ET_0_/RC was −8.13 under D, while the respective values in light treatments were between 1.32 and 1.43.

By applying statistics to all treatments (i.e., including D), no significant differences in DI_0_/RC, ABS/RC, and ET_0_/RC values were noted among seedlings exposed to B, W, RB, and R light (Figure 6I,III,V). Given the considerable difference in the values noted under D, these values were removed, and the statistical analysis was repeated (i.e., excluding D). This new analysis indicated that seedling exposure to R light led to increased DI_0_/RC, ABS/RC, and ET_0_/RC values, as compared to the other light treatments (Figure 6II,IV,VI). In addition, it was noted that seedling exposure to B light resulted in a decreased DI0/RC and ABS/RC, though the latter was not significantly different when compared to RB light.

**Figure 7:**
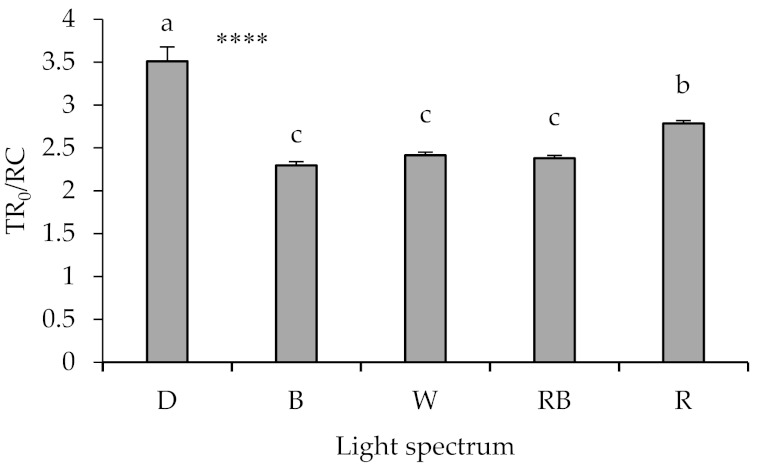
Specific energy fluxes per reaction center (RC) for trapped energy flux (TR_0_/RC; equations and explanations in Table 1), from the fluorescence transient exhibited by leaves sampled from grafted watermelon seedlings exposed for 12 days to either darkness (D) or different light quality regimes [blue (B), white (W), red and blue (RB), as well as red (R); see spectrum in Supplementary Figure S3]. Photosynthetic photon flux density was 20 ± 1 µmol m^−2^ s^−1^ during the first 2 days, and 50 ± 1 µmol m^−2^ s^−1^ during the following days. Nine plants per treatment were assessed. Different letters indicate that values are significantly different at *p* < 0.01 according to Duncan’s multiple range tests. Bars represent SEM. Significance at the 0.0001 probability level is indicated by ****.

TR_0_/RC was the highest in seedlings healed and acclimatized under D, followed by those exposed to R (Figure 7). No significant difference was noted in TR_0_/RC value between seedlings exposed to B, W, or RB light.

**Figure 8:**
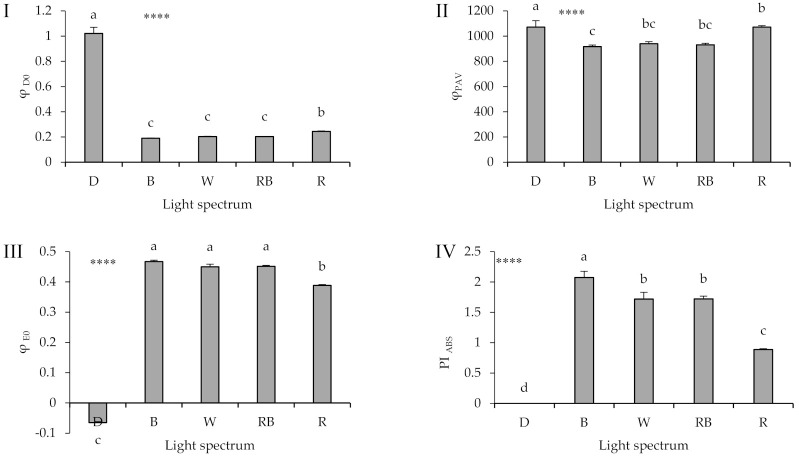
Quantum yield of energy dissipation (φ_D0_; **I**), quantum yield for primary photochemistry (φ_PAV_; **II**), quantum yield of electron transport (φ_E0_; **III**), performance index in light absorption basis (PI_ABS_; **IV**); equations and explanations in Table 2) from the fluorescence transient exhibited by leaves sampled from grafted watermelon seedlings exposed for 12 days to either darkness (D) or different light quality regimes [blue (B), white (W), red and blue (RB), as well as red (R); see spectrum in Supplementary Figure S3]. Photosynthetic photon flux density was 20 ± 1 µmol m^−2^ s^−1^ during the first 2 days, and 50 ± 1 µmol m^−2^ s^−1^ during the following days. Nine plants per treatment were assessed. Different letters indicate that values are significantly different at *p* < 0.01 according to Duncan’s multiple range tests. Bars represent SEM. Significance at the 0.0001 probability level is indicated by ****.

A striking difference between healing and acclimatization under either D or light was also noted in φ_D0_ and φ_E0_ values (Figure 8I,III; equations and explanations in Table 2). A difference between exposure to D or light treatments was also evident in φ_PAV_ parameter (equation in Table 2), though the magnitude of the effect was considerably less (Figure 8II). Among the light treatments, R-exposed seedlings showed increased φ_D0_ value, in combination with decreased φ_E0_ value.

The PI_ABS_ (equation and explanation in Table 2) was 0 in the seedlings that were exposed to D (Figure 8IV). Among the light treatments, B-exposed seedlings showed the largest PI_ABS_ value, while R-exposed plants showed the lowest one.

To facilitate the comparison of light quality regimes (i.e., B, W, RB, and R, thus excluding D), the parameters (values relative to W treatment) of the OJIP test were plotted in a spider plot (Supplementary Figure S2(II)). Light spectra caused substantial changes in the fate of the absorbed light by PSII. Seedling exposure to light containing B (i.e., B, W, and RB treatments) led to an enhanced electron transport flow, whereas monochromatic R caused an adverse effect on the photosynthetic functionality (Supplementary Figure S2(II)). Seedlings exposed to RB were comparable to the ones under the W regime, since the parameters related to fluorescence transient induction showed similar trends.

**Figure 9:**
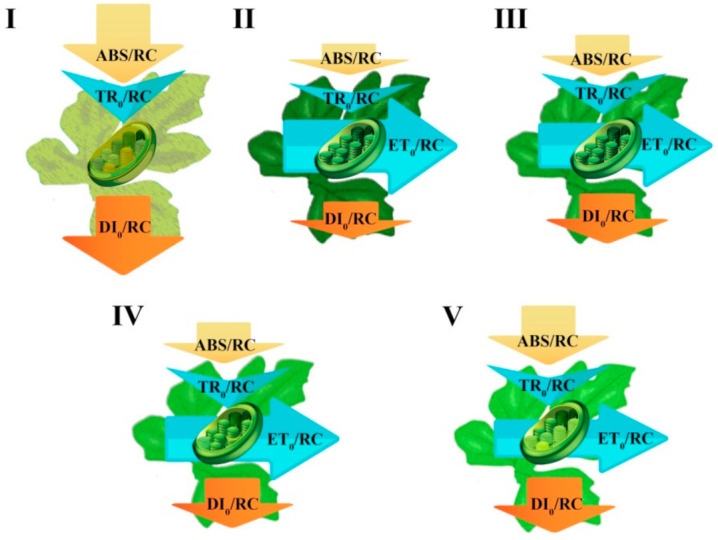
Energy pipeline models of grafted watermelon seedlings exposed for 12 days to either darkness (D; **I**) or different light quality regimes [blue (B; **II**), white (W; **III**), red and blue (RB; **IV**), as well as red (R; **V**); see spectrum and parameters’ equations in Supplementary Figure S3 and Table 2, respectively]. Photosynthetic photon flux density was 20 ± 1 µmol m^−2^ s^−1^ during the first 2 days, and 50 ± 1 µmol m^−2^ s^−1^ during the following days. The relative magnitude of each activity or fluctuation is shown by the size of the corresponding arrow. The intensity of green color of the leaves and chloroplasts illustrates their overall health functionality.

Figure 9 shows the energy pipeline model of watermelon seedlings exposed to different lighting environments, regrouping fluctuations of the specific energy parameters (i.e.; ABS/RC, TR_0_/RC, DI_0_/RC and ET_0_/RC) and the average antenna size per one active reaction center. The pale thylakoids are illustrated as heat sinks that could not absorb the light energy in the same way as active RCs do. These heat sinks are not able to store and transfer the excitation energy as redox energy in the electron transport chain; thereby, dissipating their total excitation energy as heat which is called heat-sink centers or silent centers. The greenness of the leaves demonstrate their total chlorophyll content. It is shown that the D-exposed seedlings (Figure 9I) had much less chlorophyll content accompanied by much lower electron transport, while, other seedlings especially those exposed to B-contained spectra contained more chlorophyll that was accompanied by high electron transport.

## 3. Discussion

The effect of light quality during healing and acclimatization was evaluated by examining a wide range of quality attributes in grafted watermelon seedlings.

### 3.1. Light during Healing and Acclimatization Improves Survival Ratio of the Grafted Seedlings

The role of presence or absence of light and its quality during healing (~0–7 days) and acclimatization (~8–14 days) on determining growth and vigor of grafted seedlings was investigated. Grafted seedling survival ratio was reduced to 26% under D, whereas all seedlings survived in the presence of light irrespective of its quality (Table 1). Therefore, light is essential for seedling survival during healing and acclimatization. Enhanced mortality ratio under D has also been earlier documented in tomato grafted seedling [5,19]. In other taxa, light during healing has been shown to stimulate callus induction [20], vascular connections’ formation [21] and scion-to-rootstock vascular system functioning (thus restoring water flow) [22]. The D-induced impairment of callus formation generally results in leaf shedding, and decreasing grafted seedlings’ survival [5]. The seedlings that survived after the 12-day period in D also showed inferior shoot development (indicated by both shoot dry weight and leaf number) and considerably weaker root system (indicated by both root length and dry weight), as compared to the seedlings exposed to light (Table 1). Since carbon assimilation is absent under light deprivation, plants survive by consuming the carbohydrates present in the scion [23]. Prolonged exposure to D leads to a rapid exhaustion of transitory starch reserves [24], and plants sustain metabolism by consumption of lipids and amino acids [25,26,27]. Prolonged light deprivation also disrupts developmental, transcriptional and hormonal changes, which are vital for growth [28]. Overall, the reduced (above and below ground) growth of the D-exposed seedlings in combination with their inferior quality set them as not marketable.

### 3.2. Morphology and Growth of the Grafted Seedlings Depends on the Light Spectrum during Healing and Acclimatization

Shoot dry weight and the DQI were not significantly affected by light quality during healing and acclimatization (Table 1). By examining other traits, light regime-induced differences in grafted seedling quality were clearly evident. Leaf area is an important indicator of seedling quality, and thus of marketability. Among light treatments, W-exposed seedlings had the largest leaf area as compared to seedlings exposed to the remaining light regimes, besides RB light. By contrast, R-exposed seedlings had the lowest leaf area. The difference in leaf area between W- and R-exposed seedlings was attributed to the area of individual leaves rather than their number. The increased SLA (thus thinner leaves) of W-exposed seedlings contributed to the enhanced area of individual leaves [5]. Although the reduced leaf area of R-exposed seedlings did not compromise shoot and root dry weight at this stage, it may slow down field establishment. Therefore, light quality during healing and acclimatization imposed considerable variation in seedling quality as reflected by leaf area.

LMR was affected by both presence of light and its quality; increased under D and RB and decreased under B and R, while W-exposed seedlings showed an intermediate value. The high value of LMR under D can be attributed to both decreased shoot (mostly scion) and root dry weights, while in RB-exposed seedlings the increase in LMR is mainly attributed to just decrease in root dry weight. Since differences of shoot dry weight among light treatments were not significant, the lower LMR of B- and R-exposed seedlings is the result of their increased root dry weight.

A high proportion of B in the growth spectrum (i.e., B and W) led to increased scion length as compared to the other light treatments, while scion stem diameter remained unaffected. These findings are in accordance with previous studies showing that B light generally promotes stem elongation as compared to monochromatic R light in the seedling stage [29,30,31], though in subsequent growth stages the opposite trend is often apparent [32]. In the seedling stage, the B-induced enhancement of stem elongation has been associated with increased accumulation of bioactive gibberellins, as compared to monochromatic R light [33]. The increase in seedling length without a reduction in stem diameter under the light regimes with a high proportion of B (i.e., B and W) is considered as a positive factor, enhancing seedling quality.

Root length and dry weight were significantly lower in RB-exposed seedlings, as compared to the other light quality treatments. The reduced root dry weight of these seedlings also led to increased shoot-to-root ratio. A well-developed root system and an optimum shoot-to-root ratio have been earlier suggested to be critical for successful establishment in the field [7]. Therefore, the reduced root development of RB-exposed seedlings may impose maintaining them for longer periods in the nurseries (thus increased associated costs), until roots spread over and seedlings are ready for transferring to the field. In this regard, the RB growth spectrum appears to be less advantageous for seedling healing and acclimatization, as compared to the remaining light treatments.

Regimes with increased proportion of B in the light spectrum stimulate stomatal conductance, resulting in increased gas exchange [34]. Although increased transpiration during the first days of healing (~0–4 days) is generally regarded as a negative factor and has been related to scion wilting [14], it was here counteracted by both darkness (0–2 days) and using elevated relative air humidity conditions (≥95% RH) [35,36]. Following healing, relative air humidity was gradually decreased, and the increased stomatal conductance noted under enhanced B conditions is expected to be a positive factor contributing to growth during the remaining healing and acclimatization period, through facilitating carbon dioxide intake.

Although the importance of light quality during healing and acclimatization has been earlier highlighted, different regimes have been proposed to be optimal. For instance, grafted tomato seedling quality was improved through monochromatic R light during healing and acclimatization [5]. Instead, in grafted pepper seedlings, growth was promoted by application of both B and R LEDs [6]. A combination of R and B lights was also suggested to be optimum in terms of growth in grafted watermelon seedlings [3], though different (rootstock and scion) cultivars were used as compared to our investigation. Our results and those of earlier studies [3,5,6], clearly suggest that the effects of the light quality on the grafted seedling growth appear to be not only species- but even cultivar- dependent.

Monochromatic R light during healing and acclimatization induced downward curling of leaf margins (the so-called leaf epinasty; Figure 1II), whereas monochromatic B light led to flattened leaves. Earlier studies attributed this variation in lamina curvature to differences in the epidermal cell extension among the two leaf sides, which are driven by the imbalance of metatopoline and auxin distribution across them [37,38,39]. This imbalance can be prevented by including a very low level (0.1 µmol m^−2^ s^−1^) of B in the spectrum [40,41], which promotes epidermal cell elongation on the abaxial leaf side [42]. The monochromatic R light-induced downward curling of leaf margins has been related to the reduced light interception [43]. On top of this, monochromatic R light during healing and acclimatization compromises the visual quality of the produced seedlings, by inducing a peculiar leaf form, and in this way is expected to decrease their marketability.

### 3.3. Light Spectra during Healing and Acclimatization Affects Pigment Content in Different Parts of the Seedlings

Chlorophyll concentration in different parts of the grafted seedlings is an important criterion of quality [2]. The light regime effect on photosynthetic pigment content, varied depending on the leaf type and location. D decreased rootstock cotyledon chlorophyll content, and increased both scion cotyledon and leaf carotenoid content. Earlier studies indicate that light deprivation initially stimulates chloroplast ultrastructure alterations (e.g., thylakoids become swollen), and eventually leads to chlorophyll degradation. The monochromatic B light effect was only apparent in rootstock cotyledon (higher chlorophyll and carotenoid content), whereas the effect of monochromatic R light was noted in both scion cotyledon and leaf (decreased chlorophyll content). The adverse effect of monochromatic R light on chlorophyll content is possibly related to the increased carbohydrate accumulation, since it has been associated with chloroplast deformation and depletion of chlorophyll [44,45]. Additionally, chlorophyll biosynthesis is negatively affected by B light deficiency in several taxa including spinach [46], rose [47], cucumber [29], lettuce [48] and basil [49]. Therefore, seedlings healed and acclimatized under either D or monochromatic R light are expected to have low marketability owing to reduced greening. By contrast, the grafted seedlings under monochromatic B light were found to be superior as compared to the ones exposed to either RB or W regime.

### 3.4. Photosynthetic Functionality Is Affected by the Presence of Light and Its Spectrum during Healing and Acclimatization

During healing and acclimatization, functional photosynthesis is essential for the overall quality of grafted seedlings, determining their marketability. The functionality of photosynthetic apparatus was assessed by both F_v_/F_m_ imaging and OJIP test. Both methods pointed out a strong negative effect of D on photosynthetic performance. The decrease in PSII activity, owing to D exposure, has been correlated to chlorophyll degradation and ultrastructure alteration [50]. Therefore, D-exposed seedlings not only have lower chlorophyll content but also the remaining chlorophyll could facilitate photosynthesis in a less efficient manner.

Healing and acclimatization under D also led to an increase in the parameters related to the energy dissipation in PSII [51], such as DI_0_/RC and Φ_D0_ (Figure 6III and Supplementary Figure S2(I)). DI_0_/RC is related to partial deactivation of PSII reaction centers, and is generally increased when these centers cannot transfer energy upstream of PSII owing to thylakoid membrane damage. A high rate of DI_0_/RC as compared to the corresponding ET_0_/RC value is most probably associated with the reduced F_v_/F_m_ value and a higher chance of photoinhibition occurrence. This is because a reduction in F_v_/F_m_ value generally occurs when the PSII function and structure are damaged by stress, causing most of the light energy absorbed to be dissipated from the PSII reaction center. Following 14 days exposure to D, the light energy absorbed by one active reaction center (ABS/RC) was dramatically higher. This striking increase may be the result of either PSII response centers’ inactivation or receptors’ size increase. Additionally, the energy used to reduce Q_A_ by the RC unit of PSII (TR_0_/RC) was the highest in grafted seedlings under D. The effect of D on overall PSII photosynthetic activity is also clearly manifested by examining PI_ABS_ (Figure 8IV), which is the most sensitive OJIP-test parameter. A decrease in PI_ABS_ value is generally associated with a lower capacity for development of the trans-thylakoid proton gradient. The decrease in PI_ABS_ also indicates that the system structure, potential activity of PSII, damage/repair ratio of protein D_1_ of PSII may be damaged or unable to completely advance under certain light conditions. Short (24 h) exposure to D already caused a drastic (64%) F_v_/F_m_ decrease in *Asterochloris erici* [52]. Prolonged light deprivation has been earlier associated with attenuated activity of PSI, PSII, cytb_6_f and ATP synthase in *Arabidopsis thaliana* [53]. The high F_0_ intensity of dark-developed chloroplasts is attributed to an enhanced fraction of closed PSII core, which disturbs PSII activity and electron transport [54,55]. Among light treatments, monochromatic R light significantly down-regulated the biophysical parameters related to the PSII efficiency such as F_v_/F_0_, F_m_/F_0_, and F_v_/F_m_, which has been associated to either damage or long-term down-regulation of PSII owing to stress [38]. This negative effect of monochromatic R light has also been noted in other taxa (the so-called red light syndrome; [38,39,56], though species not undergoing any adverse effect have also been referred [57]. Although the processes underlying the attenuated PSII efficiency in R-exposed plants have not been clarified, impaired PSII core proteins’ synthesis, D_1_ protein damage and PSII reaction centers’ inactivation have been implicated as contributing factors [38,49].

Seedling growth under monochromatic R light led to an effect on the parameters related to the energy dissipation in PSII such as DI_0_/RC and Φ_D0_, which was similar to D in terms of direction, but at smaller magnitude. Similar results have been reported for three pot plants exposed to monochromatic R light [58]. The increased ABS/RC under monochromatic R light has been related to the reduction in the number of active PSII reactive centers, which might serve as a defense mechanism to alleviate the light stress pressure on the photosynthetic system [59]. The adverse effect of monochromatic R light on overall PSII photosynthetic activity is also evident by examining the PI_ABS_ parameter.

The highest soluble carbohydrate content was noted in R-exposed seedlings. An intermediate one was noted under RB light, while the lowest was noted in the regimes with a high proportion of B (i.e., B and W). The same trend has been earlier noted in lettuce leaves [11]. The monochromatic R light effect on photosynthetic product accumulation has been validated in several taxa. Since both auxin biosynthesis and transport are affected by carbohydrate content, the latter may underlie the noted leaf epinasty under R light [60]. Although carbohydrate synthesis is reduced owing to disturbed photosynthetic apparatus, the increased carbohydrate content in R-exposed seedlings is possibly the result of reduced translocation out of the leaves [61]. This increased carbohydrate accumulation has been associated with both down-regulation of photosynthesis [62] and feedback inhibition on sucrose synthesis and sugar phosphates’ cytosol accumulation [63]. Since B light generally up-regulates the genes encoding key enzymes in the Calvin-Benson cycle, feedback control is ruled out in the inhibition of photosynthesis under monochromatic B light [63,64].

### 3.5. High Proportion of B (i.e., B, W, RB) in the Overall Spectrum Up-Regulates Electron Transport, Leading to Improved Photosynthetic Functionality

Several parameters of both employed methods (F_v_/F_m_ imaging and OJIP test) pointed out a superiority of seedlings that were exposed to B light. Among light treatments, B light led to the highest values in F_v_/F_0_, F_v_/F_m_, as well as F_m_/F_0_, and the lowest value in DI_0_/RC. This enhancement following exposure to monochromatic B light is also reflected by the PI_ABS_ parameter. In these parameters (F_v_/F_0_, F_v_/F_m_, F_m_/F_0_, DI_0_/RC, and PI_ABS_), no significant difference was noted between seedlings exposed to W or RB light. Therefore, B-exposed seedlings have not only elevated chlorophyll content (Figure 3I), but also chloroplasts are able to perform photosynthesis in a more efficient context, as compared to the other light quality regimes.

Treatments containing high proportion of B (i.e., B, W, RB) had a decreased energy absorption per active RC but overall increased electron transport, due to more active RCs (Figure 9). Dissipation occurs as heat, fluorescence and energy transfer to other systems. It is also influenced by the ratios of active/inactive RCs. ABS/RC in D-exposed seedlings increased mainly due to the impairment of the RCs. Among light-exposed seedlings, the same increase (but with lower magnitude) happened for R-exposed seedlings. D-exposed seedlings also revealed an increased ratio of total dissipation to the amount of active RCs (DI_0_/RC) mainly due to the high dissipation of the inactive RCs.

Although the LED efficiency is determined by several factors, a photon at shorter wavelengths (i.e., B) fundamentally carries more energy, as compared to one at longer wavelengths. This increased energy per photon in the B region impedes the B LEDs’ efficiency. Another downside of applying monochromatic B light in practice is the difficulty of detecting both nutrient deficiency and disease symptoms. That said, W light appears to be the best alternative. Although most of the assessed traits were similar between exposure to W or RB light, W light better stimulated scion length and root growth.

## 4. Materials and Methods

### 4.1. Plant Material and Exposure Conditions

A commercial watermelon cultivar [*Citrullus lanatus* (Thunb.) Matsum. and Nakai. cv. Crimson] and a commercial rootstock cultivar [*Cucurbita maxima* Duchesne ex Poir. × *Cucurbita moschata* (Duchesne ex Lam.) cv. Marvel] were employed. This rootstock was selected as it is frequently used in commercial practice in Iran due to its compatibility with melon, and its tolerance to low temperature and soilborne pathogens. Graded seeds of scion and rootstock were soaked in distilled water at room temperature (25 °C), a day prior to sowing. To produce seedlings with approximately the same size and diameter of hypocotyls, the scion seeds were sown 7 days earlier than the rootstock seeds. This was done in 50-cell trays filled with a commercial growing mixture containing peatmoss, perlite and cocopeat in a ratio of 7:2:1 (*v*/*v*/*v*). The trays were placed in a glass-covered greenhouse (Karaj, 35°51′21’’ N), where day/night temperature was controlled to 26/18 °C, and photoperiod to 12 h.

When the scion and rootstock cotyledons had fully opened, one-cotyledon grafting was conducted. In rootstock, one cotyledon along with the visible growing point were removed (45° angled cut) by using a semi-automatic grafting robot (model type GR 300/3; Atlantic Man s.r.l., Castelnovo di Sotto, Italy). Thereafter, the scion (bearing two cotyledons) was placed on the rootstock hypocotyl by using a plastic clip. In this step, the rootstock roots were removed to prevent water accumulation in the graft union, owing to enhanced (rootstock) root pressure.

Grafted seedlings were then transferred to a specialized healing room equipped with an automatically-controlled air conditioning system. These were initially exposed to darkness (D) for 2 days to prevent leaf dehydration. Then, the seedlings were placed in five healing and acclimation cabinets (LED-equipped vertical systems with three floors; Supplementary Figure S3), each under different light regime, including B (peak at 460 nm), white [W; 35% blue (400–500 nm), 49% intermediate (500–600 nm), and 16% red (600–700 nm)], R (68%) and B (RB), as well as R (peak at 660 nm; Supplementary Figure S3). Light was provided by LED modules (Iraneon Co, Birjand, South Khorasan, Iran), and spectra were monitored using a Sekonic light meter (Sekonic C-7000, Tokyo, Japan). In all four healing and acclimation cabinets with illumination, light intensity was adjusted to 20 ± 1 µmol m^−2^ s^−1^ photosynthetic photon flux density (PPFD) at the top of the plant canopy for 2 days, and then to 50 ± 2 µmol m^−2^ s^−1^ PPFD for the next 10 days. All plants were exposed to the same controlled conditions, i.e., 16 h photoperiod, and 25/20 °C day/ night air temperature. Relative air humidity was gradually decreased, being 95–98% (Days 0–2), 85–90% (Days 3–4), 80–85% (Days 5–7), and 60–70% (Days 8–14). Growth-media moisture was maintained at or near maximum water holding capacity by regular watering.

Nine plants were sampled (three per vertical system floor) per light regime. To minimize border effects, these were surrounded by border plants (periphery of the tray) that were not sampled. After 14 days in the healing and acclimation cabinets, measurements were performed. Sampled leaves were young, fully-expanded, and grown under direct light. In all cases, the time between sampling and the start of the evaluation did not exceed 15 min.

### 4.2. Survival Ratio, Plant Biomass and Leaf Morphology

The effect of light regime on seedling survival, growth and morphology was assessed. The survived seedlings relative to the ones that were initially placed (the so-called survival ratio) was calculated. Evaluations also included rootstock length (from the root-to-shoot junction to the graft union), scion length (from the graft union to the apical meristem), scion diameter (1 cm above the graft union), number of leaves, leaf area, leaf and aboveground dry masses. For measuring dry weight, samples were placed in a forced-air drying oven for 72 h at 80 °C [65]. For leaf area assessment, leaves were scanned (HP Scanjet G4010, Palo Alto, CA, USA) and then evaluated by using the Digimizer software (version 4.1.1.0, MedCalc Software, Ostend, Belgium). Before scanning, curled leaves (noted under monochromatic R light) were flattened out on a white paper. In this way, the leaf surface curvature-induced reduction in projected area was minimized. Thereafter, the specific leaf area (SLA; leaf area/leaf dry mass), and leaf mass ratio (LMR; leaf dry mass/plant dry mass) were calculated [66]. Following removal of the substrate from the roots via gentle washing, root length and dry mass were also measured.

$$\mathrm{The}\;\mathrm{Dickson’s}\;\mathrm{quality}\;\mathrm{index}\;(\mathrm{DQI}=\frac{\mathrm{Seedling}\;\mathrm{total}\;\mathrm{dry}\;\mathrm{weight}\;\left(g\right)}{\frac{\mathrm{Seedling}\;\mathrm{height}\;\left(\mathrm{mm}\right)}{\mathrm{Scion}\;\mathrm{Stem}\;\mathrm{diameter}\;\left(\mathrm{mm}\right)}+\frac{\mathrm{Shoot}\;\mathrm{dry}\;\mathrm{weight}\;\left(g\right)}{\mathrm{Root}\;\mathrm{dry}\;\mathrm{weight}\;\left(g\right)}})$$

was also calculated. This index is commonly employed for the evaluation of tree seedlings’ quality, and it has also been recently introduced for assessing the quality of grafted vegetable seedlings [2].

Prior to biomass evaluation, plant images were also obtained by using a digital camera (Canon EOS M2; Canon Inc., Tokyo, Japan). The captured images allowed the evaluation of plant morphology. The plants were manually moved to the image capture station. The imaging station included top and side lighting units (fluorescent tubes; Pars Shahab Lamp Co., Tehran, Iran). A side view perspective (perpendicular to the shoot axis) was employed [67]. The camera-to-plant distance was maintained at 1 m. One image (RGB) was obtained per plant (including shoot, one representative leaf, and root), and nine plants (three per vertical system floor) were imaged per treatment.

### 4.3. Determination of Soluble Carbohydrates

Leaf samples were ground in liquid nitrogen, and 0.2 g tissue was sampled and blended with 7 mL of 70% ethanol (*w*/*v*) for 5 min on ice and centrifuged (6700× *g*) for 10 min at 4 °C. After adding 200 mL of the supernatant to 1 mL of an anthrone solution (0.5 g anthrone, 250 mL 95% H_2_SO_4_, and 12.5 mL distilled water), the absorbance was spectrophotometrically (UV-1800, Shimadzu, Japan) recorded at 625 nm. Nine scion leaves (three per vertical system floor) were assessed per treatment. Replicate leaves were collected from separate plants.

### 4.4. Photosynthetic Pigments

The light regime effect on photosynthetic pigment (chlorophyll, carotenoids) content was assessed in rootstock cotyledon, scion cotyledon, and scion leaf. Leaf samples were processed immediately after collection. Following fine chopping, portions weighing 0.5 g were homogenized with the addition of 10 mL of 80% acetone. This primary acetone extract was then filtered, and the filtered extract was diluted by adding 2 mL of 80% acetone per mL of extract. Since chlorophyll is light sensitive, extraction took place in a dark room [68]. The obtained extract was subjected to reading on a spectrophotometer (Mapada UV-1800; Shanghai. Mapada Instruments Co., Ltd., Shanghai, China). Total chlorophyll and carotenoid contents were calculated [69]. Nine leaves (three per vertical system floor) were assessed per treatment. Replicate leaves were collected from separate plants.

### 4.5. Chlorophyll Fluorescence Imaging

As a sensitive indicator of plant photosynthetic performance, dark-adapted values of the maximum quantum yield of PSII (F_v_/F_m_; equation in Table 2) were recorded in leaves detached from plants of each light regime. Measurements were conducted on leaves by using a FluorCam FC 1000-H (Photon Systems Instruments, Drásov, Czech Republic). By turning LEDs off, the leaves were dark adapted (≥20 min) prior to evaluation. Then, F_v_/F_m_ was evaluated by applying a saturated PPFD of 3900 µmol m^−2^ s^−1^ [17,70,71]. Nine scion leaves (three per vertical system floor) were assessed per treatment. Replicate leaves were collected from separate plants.

### 4.6. Polyphasic Chlorophyll Fluorescence Transient (OJIP) Evaluation

A polyphasic chlorophyll fluorescence induction curve (O–J–I–P-transient) was obtained in leaves attached to plants of each light regime. By employing the OJIP test, the shape changes of the OJIP transient are quantitatively translated to a set of parameters (equations and explanations in Table 2), which relate to the in vivo adaptive behavior of the photosynthetic apparatus (especially PSII) to the growth environment [70,72]. Measurements were conducted on attached leaves by using a PAR-fluorPen FP 100-MAX (Photon Systems Instruments, Drásov, Czech Republic) following dark adaptation (≥20 min). The employed light intensity (3900 μmol m^−2^ s^−1^ PPFD) was sufficient to generate maximal fluorescence for all light quality treatments.

Following dark adaptation, leaves exhibit a polyphasic chlorophyll fluorescence rise during the first second of illumination. The fluorescence transient, plotted on a logarithmic time scale, typically includes the following phases: O to J, J to I, and I to P. F_0_ represents the so-called “open” (O) state of the O–J–I–P-transient [73] measured at 50 µs. For non-stressed plants, the F_0_ value drastically differs, and thus a simple range cannot be reported. Yet, higher F_0_ values indicate exposure to more intense stress. F_0_ primarily originates from the light-harvesting antenna pigments [74,75]. F_j_ and F_I_ originate from the inflections at 2 and 30 ms, respectively [76]. F_m_, on the other hand, comes from the reduction–oxidation state of the primary quinone electron acceptor of PSII (Q_A_). The highest F_v_/F_m_ value is around 0.84 for horticultural plants, while values higher than 0.8 are often detected in healthy plants. φ_E0_ is a metric of the quantum yield of the electron transport from Q_A_- to plastoquinone (PQ), and drops under stress conditions [77]. Overall, decreasing values of F_v_/F_0_, F_v_/F_m_, F_m_/F_0_, φ_E0_, and PI_ABS_ indicate stress *in planta* [75,76]. Nine scion leaves (three per vertical system floor) were assessed per treatment. Replicate leaves were collected using separate plants.

**Table 2:** Abbreviations, definitions, and formulas of the O–J–I–P parameters assessed in the current study.

Basic Parameters		
**F_0_**	Minimum fluorescence, when all PSII reaction centers (RCs) are open (O-step of OJIP transient)	F_50µs_
**F_J_**	Fluorescence intensity at the J-step (2 ms) of OJIP	F_2 ms_
**F_I_**	Fluorescence intensity at the I-step (30 ms) of OJIP	F_30ms_
**Fluorescence Parameters**		
**F_m_**	Maximum fluorescence, when all PSII RCs are closed (P-step of OJIP transient)	F_1s_ = F_p_
**F_v_**	Variable fluorescence of the dark-adapted leaf	F_m_ − F_0_
**V_J_**	Relative variable fluorescence at time 2 ms (J-step) after start of actinic light pulse	(F_J_ − F_0_)/(F_m_ − F_0_)
**V_I_**	Relative variable fluorescence at time 30 ms (I-step) after start of actinic light pulse	(F_30ms_ − F_0_)/(F_m_ − F_0_)
**F_v_/F_m_**	Maximal quantum yield of PSII photochemistry	1 − (F_0_/F_m_) = (F_m_ − F_0_)/F_m_ = φ_P0_ = TR_0_/ABS
**Quantum Yields and Efficiencies/Probabilities**		
**φ_E0_**	The quantum yield of electron transport	[1 − (F_0_/F_m_)](1 − V_J_) = ET_0_/ABS
**φ_D0_**	Quantum yield of energy dissipation	F_0_/F_m_
**φ_PAV_**	Average quantum yield for primary photochemistry	φ_P0_ (1 − V_J_) = φ_P0_ (S_M_/t_FM_)
**Specific Energy Fluxes** (**Per Q_A_ Reducing PSII RC**)		
**ABS/RC**	The specific energy fluxes per RC for energy absorption	M_0_ (1/V_J_)(1/φ_P0_)
**TR_0_/RC**	Trapped energy flux (leading to Q_A_ reduction) per RC	M_0_ (1/V_J_)
**ET_0_/RC**	Electron transport flux (further than Q_A_^−^) per RC	M_0_ (1/V_J_)(1 − V_J_)
**DI_0_/RC**	Dissipated energy flux	(ABS/RC) − (TR0 /RC)
**Performance Indexes** (**Products of Terms Expressing Partial Potentials at Steps of Energy Bifurcations**)		
**PI_ABS_**	Performance index for the photochemical activity	[(γRC/1 − γRC) (φ_P0_ /1 − φ_P0_) (ψ_E0_ /1 − ψ_E0_)]

### 4.7. Energy Pipeline Model

To study the mechanisms of photosynthesis in each treatment, the so-called energy pipeline model has been developed and illustrated (Figure 9). Energy pipeline is a dynamic model in which the value of each energy flux, here, modified by the different imposed lighting conditions, which is expressed by the appropriately adjusted size of the corresponding arrow. In general, two types of models can be presented, one of which refers to the RC in the membrane and thus deals with the specific energy fluxes per RC and the other refers to the excited cross-section (CS) of a leaf, thereby dealing with the phenomenological energy fluxes per CS. In the present study, we developed the former one (i.e., per RC; membrane model). The membrane energy pipeline model includes also a demonstration of the average “antenna size”, which follows the value of the ABS/RC. This value expresses the total absorption of PSII antenna chlorophylls divided by the number of active (in the sense of Q_A_ reducing) reaction centers.

### 4.8. Statistical Analysis

The experiments were arranged in a completely randomized design. Data were analyzed using SAS software (v. 9.4, SAS Institute Inc., Cary, NC, USA). Mean separations were calculated using Duncan’s multiple range test at *p* ≤ 0.05.

## 5. Conclusions

The effect of light and its quality during healing and acclimatization of grafted watermelon seedling was assessed. Seedling survival was strongly decreased by D condition. The seedlings that survived showed inferior shoot development, weaker root system, reduced (rootstock cotyledon) chlorophyll content, and reduced photosynthetic apparatus functionality (assessed by both F_v_/F_m_ imaging and OJIP test). Seedling survival was 100% under all light regimes irrespective of their quality. Shoot dry weight and the DQI were not illustrative in quantifying variation in grafted seedling quality among light quality treatments. Although W and RB light-produced seedlings were similar in the majority of the assessed traits, W light better promoted seedling scion length and root growth. RB-exposed seedlings had a less-developed root system, requiring more time to produce marketable plants. R-exposed seedlings had the smallest leaf area, and underwent leaf epinasty, which not only compromises visual quality, but also reduces light interception. Seedlings that were exposed to monochromatic R light showed reduced (scion cotyledon and leaf) chlorophyll content, limiting their subsequent marketability. The photosynthetic apparatus functionality was also suppressed in R-exposed seedlings, as compared to the remaining light treatments. B-exposed seedlings had advantageous shoot morphology (elongated scion, flattened leaves), the highest (rootstock cotyledon) chlorophyll content, and could facilitate photosynthesis in a more efficient manner.

## Figures and Tables

**Table 1:** Growth and morphology of grafted watermelon seedlings exposed for 12 days to either darkness (D) or different light quality regimes [blue (B), white (W), red and blue (RB), as well as red (R); see spectrum in Supplementary Figure S3]. Photosynthetic photon flux density was 20 ± 1 µmol m^−2^ s^−1^ during the first 2 days, and 50 ± 1 µmol m^−2^ s^−1^ during the following days. Nine plants per treatment were assessed. Means within a column followed by the same letters are not significantly different at *p* ≤ 0.05 according to Duncan’s multiple range test.

Light Regime	Survival Ratio (%)	Rootstock Length (cm)	Scion Length (cm)	Scion Stem Diameter (cm)	Leaf Number (plant^−1^)	Leaf Area (cm^2^ leaf^−1^)	Leaf Area (cm^2^ plant^−1^)	Plant Dry Weight (g)	Shoot Dry Weight (g)	Specific Leaf Area (SLA; cm^2^ g^−1^)	Leaf Mass Ratio (LMR; g g^−1^)	Root Length (cm)	Root Dry Weight (g)	Shoot to Root Ratio (g/g)	Dickson’s Quality Index (DQI)
**D**	26.3 ^b^	7.51	2.45 ^b^	0.31	2.3 ^c^	5.01 ^d^	26.4 ^d^	0.174 ^b^	0.165 ^b^	247 ^c^	0.38 ^a,b^	4.14 ^b^	0.009 ^c^	19.17 ^a^	0.006 ^b^
**B**	100 ^a^	5.87	4.15 ^a^	0.33	5.0 ^b^	8.24 ^b^	65.9 ^b^	0.290 ^a^	0.265 ^a^	417 ^b^	0.35 ^c^	6.38 ^a^	0.025 ^a^	10.53 ^b^	0.015 ^a^
**W**	100 ^a^	6.05	4.10 ^a^	0.38	5.0 ^b^	10.11 ^a^	80.8 ^a^	0.251 ^a^	0.230 ^a^	540 ^a^	0.37 ^b^	6.33 ^a^	0.021 ^a^	11.02 ^b^	0.013 ^a^
**RB**	100 ^a^	6.27	2.42 ^b^	0.40	6.0 ^a^	8.15 ^b^	73.4 ^a,b^	0.282 ^a^	0.270 ^a^	411 ^b^	0.39 ^a^	4.24 ^b^	0.012 ^b^	23.17 ^a^	0.013 ^a^
**R**	100 ^a^	6.38	2.25 ^b^	0.38	5.3 ^b^	6.28 ^c^	52.2 ^c^	0.265 ^a^	0.256 ^a^	376 ^b^	0.34 ^c^	6.72 ^a^	0.024 ^a^	10.16 ^b^	0.016 ^a^
***p***	<0.0001	0.0963	0.0075	0.23	<0.0001	<0.0001	<0.0001	0.0003	<0.0001	<0.0001	<0.0001	0.0065	<0.0001	<0.0001	0.0009

## Data Availability

The data presented in this study are available on request from the corresponding authors. The data are not public.

## Supplementary Materials

Supplementary Materials can be found at https://www.mdpi.com/article/10.3390/ijms22158043/s1.

## Abbreviations

B	blue
D	darkness
DQI	Dickson’s quality index
LMR	leaf mass ratio
O–J–I–P	polyphasic chlorophyll fluorescence induction curve
PPFD	photosynthetic photon flux density
PSII	Photosystem II
R	red
RB	red and blue
RC	reaction center
SLA	specific leaf area
W	white
