# Supplementary material for: Blue Light Improves Photosynthetic Performance during Healing and Acclimatization of Grafted Watermelon Seedlings
Source: Int J Mol Sci. 2021 Jul 28;22(15):8043. doi: 10.3390/ijms22158043 (PMC8347074; doi:10.3390/ijms22158043)
Supplement: Supplementary file 1 [file ijms-22-08043-s001.zip › ijms-1259243-supplementary.pdf]

Supplementary Information

Blue light improves photosynthetic performance during healing and acclimatization of grafted watermelon seedlings

Moein Moosavi-Nezhad, Reza Salehi, Sasan Aliniaeifard, Georgios Tsaniklidis, Ernst J. Woltering, Dimitrios Fanourakis, Krystyna Żuk-Gołaszewska, and Hazem M. Kalaji

Supplementary Figures

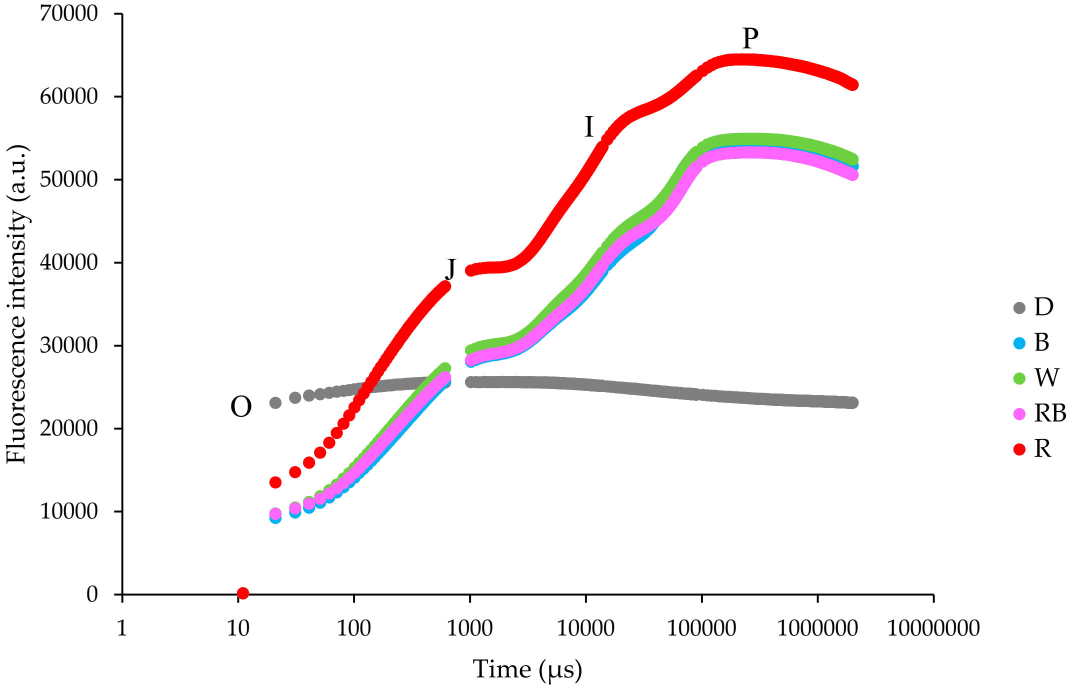

Figure S.1. Transient chlorophyll fluorescence (OJIP) of leaves sampled from grafted watermelon seedlings exposed for 12 days to either darkness (D) or different light quality regimes [blue (B), white (W), red and blue (RB), as well as red (R); see spectrum in Supplementary Figure S.3]. Photosynthetic photon flux density was 20 ± 1 μmol m^{-2} s^{-1} during the first 2 days, and 50 ± 1 μmol m^{-2} s^{-1} during the following days. Nine plants per treatment were assessed.

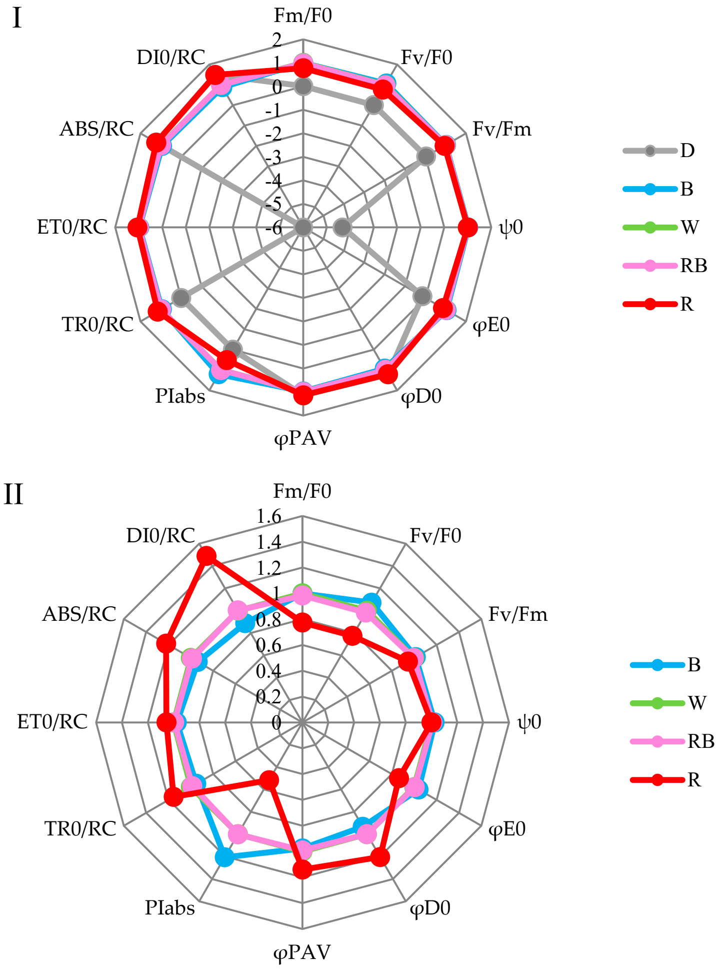

Figure S.2. Spider plot of the OJIP test parameters from the fluorescence transient (equations and explanations in Table 2) exhibited by leaves sampled from grafted watermelon seedlings exposed for 12 d to either darkness (D) or different light quality regimes [blue (B), white (W), red and blue (RB), as well as red (R); see spectrum in Supplementary Figure S.3]. Photosynthetic photon flux density was 20 ± 1 μmol m^{-2} s^{-1} during the first 2 d, and 50 ± 1 μmol m^{-2} s^{-1} during the following days. Panel II represents the same data as panel I, excluding the D treatment. The values of the calculated parameters were shown relative to those of the W treatment. Differences in the scale among panels ought to be noted.

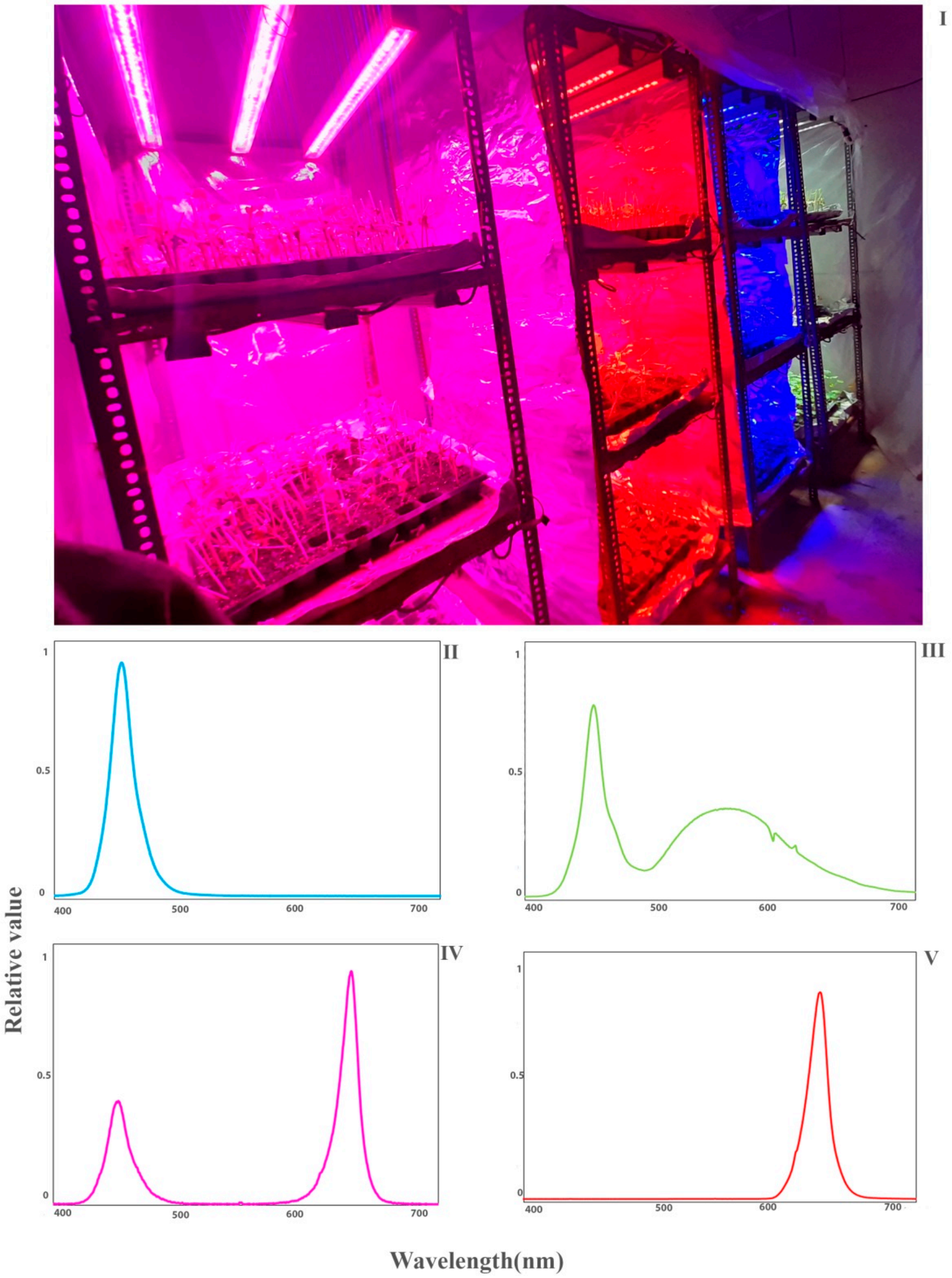

Figure S.3. Healing and acclimatization cabinets (from left to right: red and blue, red, blue, white; I), as well as spectra of blue (II), white (III), red and blue (IV), as well as red (V) light quality regimes under study. The cabinets, where no light was provided, are not shown.
